# 2024 Acknowledgment of *Microbiology Spectrum Ad Hoc* Reviewers

**DOI:** 10.1128/spectrum.02609-24

**Published:** 2024-12-05

**Authors:** Christina A. Cuomo

**Affiliations:** 1 Department of Molecular Microbiology and Immunology, Brown University, Providence, Rhode Island, USA

## EDITORIAL

The editors of *Microbiology Spectrum* sincerely thank all the reviewers who contributed to the peer review process during the period of November 2023 through October 2024. We are grateful for the contributions of almost 3,000 reviewers who have contributed their time, effort, and expertise to the journal. We would like to specifically recognize the following *ad hoc* reviewers who have reviewed three or more articles submitted to *Microbiology Spectrum*. Your dedication to the journal and its success is commendable and greatly appreciated.

Ali H. Abbas

Ghulam Abbas

Firouz Abbasian

Hamad A. Abdelhadi

Sahar M. Abduladheem

Nena Abdul

Asra'a Abdul-Jalil

Huda S. Abdul-Mohammed Al-Rawazq

Debarun Acharya

Wioletta Adamus-Białek

Kamoru A. Adedokun

Adetomiwa A. Adeniji

Cynthia A. Adinortey

Innocent Afeke

Muhammad Afzal

Genesis Julyus T. Agcaoili

Leopoldo Aguilar Faisal

Claudio Aguilar

Amin Akbari Ahangar

Anum A. Ahmad

Takanori Akase

Robert A. Akins

Michael O. Akintubosun

Pierre-Marie Akochy

Laith K. Al-Ani

Katie J. Aldred

Alison F. Alencar Chaves

Jose Alexander

Hawraa Natiq K. Al-Fatlawy

Halah H. Al-Haideri H

Hafidha S. Al-Hattali

Inas M. Alhudiri

Majid Ali Shah Akhun

Sahar A. Allam

Hassan M. Al-Tameemi

Samuel Alvarez-Arguedas

Lysangela R. Alves

Fairoz A. Al-Wrafy

Nana Ama Amissah

Neil W. Anderson

David R. Andes

Julien Andreani

Sam R. Andrews

Francescopaolo Antonucci

Alix Augusto Armero Villanueva

Eurico Arruda

Djandan Tadum Arthur Vithran

Asaad M. Ataa

Juan A. Ayala

Joseph A. Ayariga

Malik Aydin

Yong-Sun Bahn

Ying Bai

Padmapriya P. Banada

Rakshya Baral

Edwin Barrios-Villa

Sreekanth Reddy Basireddy

Sarah R. Beattie

Sujit K. Behera

Margo Bell

Jose A. Bengoechea

Fabian Berger

Sutonuka Bhar

Yogendra Bhaskar

Somanon Bhattacharya

Micah M. Bhatti

Gabriele Bianco

Xiaoli Bing

Fabiana Bisaro

Karishma Bisht

Matthew G. Blango

Karin Blomqvist

Jeffrey L. Bose

Ignacio G. Bravo

Antoinette Brink

Timmie A. Britton

Tina I. Bui

Chane Buys

Feng Cai

Alexandra Calle

Danielle E. Campbell

Andrés Carrazco Montalvo

Monica Cartelle Gestal

Vida Časaitė

Yormaris Castillo

Fatma T. Çetin

Kaan Çeylan

Benjamin J. Chadwick

Clarence W. Chan

Ioanna Chatzigiannidou

Neeraj Chauhan

Chiliang Chen

Jun Chen

You-Hee Cho

Rounak Chourasia

Andrew E. Clark

Joseph M. Colacino

James Collins

Doug Cossar

Scott Cunningham

Ram Hari Dahal

Ligia V. Dasilva

Justine W. Debelius

Maurizio Del Poeta

Wayne Xianding Deng

Justen T. Despain

Alexandre Dias Tavares Costa

Francisco Diez-Gonzalez

Bauke W. Dijkstra

Marcia Dinis

Yohei Doi

Jingjie Du

Rachael Terumbur Duche

Edward G. Dudley

Chetna Dureja

Allison R. Eberly

Tom Edlind

Eman A. Elmansoury

Andrew J. Fabich

Fernando Ferreira

Richard L. Ferrero

Jonathan G. Frye

Michael G. Ganzle

Purushottam V. Gawande

Jennifer Geddes-McAlister

Vinoj Gopalakrishnan

Stephen V. Gordon

Amanda M. Graves

Francois Gravey

Jonathan M. Greenberg

Allison Groseth

Bianli Gu

François Guérin

Xu G. Guo

Manoj Gurung

Maria Hadjifrangiskou

Sohei Harada

Robert D. Hardy

Fahim Hayat

Zilong He

Xianteng Hou

Gongzheng Hu

Ching-Ying Huang

Jinhu Huang

Peng Huang

Rongzheng Huang

Shi Huang

Jonathan D. Hulse

Tuanh N. Huynh

Ikbal Agah Ince

Hidemasa Izumiya

Avijeet S. Jaswal

Francis E. Jenney

Xinmiao Jia

Xiaoyuan Jiang

Yong Jiang

Jason T. Kaelber

Sarah E. Kidd

Kalmia E. Kniel

Hisayuki Komaki

Lukasz Kozubowski

Ranjeet Kumar

Kanupriya Kusumakar

Sebla B. Kutluay

Emily Lou Lamartina

Shuai Le

John A. Lednicky

Kyle R. Leistikow

Jonathan Lellouche

Michael J. Lemke

Shawn Lewenza

Cuidan Li

Guobang Li

Ruichao Li

Xian-Zhi Li

Yuehong Li

Yutang Li

Steven E. Lindow

Bo Liu

Dongxiao Liu

Yong-Xin Liu

Marta Lourenço

Lingna Lv

Michael Macgregor-Fairlie

Mark J. Mandel

Melissa J. Martin

Yasufumi Matsumura

Molly A. Matty

Juliana Menezes

Yixuan Meng

Aaron P. Mitchell

Maria F. Mojica

Kai Mu

Bharath Mulakala

David R. Murdoch

Shannon Murphy

Mathenge J. Mutiso

Kevin A. Nash

Debasis Nayak

Kenneth W. Nickerson

Tomoki Nishioka

Andrew P. Norgan

Dennis Nurjadi

Sheila F. O'Brien

Kazuhiro Ogai

Randall J. Olsen

Teresa R. O'Meara

Daniel A. Ortiz

Kenji Ota

Erdal Özbek

Yuanlong Pan

Sneh L. Panwar

Costas C. Papagiannitsis

Catalina Pardo-Roa

James A. Parejko

Tanya Parish

Ruvini U. Pathirana

Andrew Pekosz

Trinh C. Phan

Ansgar Poetsch

Mario Poljak

Praveen Rahi

Ashok K. Reddy

Johanna Rhodes

Charles A. Rivers

Nicole Robbins

Daniel D. Rockey

Miriam Rodriguez Fernandes

Shivaprakash M. Rudramurthy

Mustafa Sadek

Mustafa K. Şahin

Vishnu Samara

John C. Samuelson

Gary Sawers

Patrick M. Schlievert

Susan Schlimpert

Bryan H. Schmitt

Umender Sharma

Allyson E. Shea

Juntao Shen

Zeyang Shen

Wenyu Shi

Lynn L. Silver

Vijay Singh

David Šmajs

Kenneth P. Smith

K. Sreenath

Yankuo Sun

Zhihong Sun

Yuki Takamatsu

Abraham Terna Lan

Anna D. Tischler

Xinzhao Tong

Chi-Ching Tsang

Christine Y. Turenne

Katharine Uhteg

Norman Van Rhijn

Branislav Vecerek

Alejandro J. Vila

Huanyu Wang

Xin Wang

Dona Saumya S. Wijetunge

Jay N. Worley

Chenggang Wu

Wanying Wu

Li Xiao

Abbas Yadegar

Masaki Yamamoto

Jun Yang

Qian Yu

Xia Yu

Yunsong Yu

Robert Zarnowski

Melissa L. Zastrow

Lin Zeng

Lingdi Zhang

Long-Xian Zhang

Yibei Zhang

Dongchi Zhao

Jian Zhou

Lifeng Zhu

Sara L. Zimmer

Zhenqiang Zuo

